# Robot-assisted total knee arthroplasty improves mechanical alignment and accuracy of component positioning compared to the conventional technique

**DOI:** 10.1186/s40634-022-00546-z

**Published:** 2022-10-28

**Authors:** Chang Hyun Nam, Su Chan Lee, Jin-Hong Kim, Hye Sun Ahn, Ji-Hoon Baek

**Affiliations:** Joint & Arthritis Research, Department of Orthopaedic Surgery, Himchan Hospital, 120, Sinmok-Ro, Yangcheon-Gu, Seoul, 07999 Republic of Korea

**Keywords:** Robot-assisted, Total knee arthroplasty, MAKO, Polyethylene liner thickness

## Abstract

**Purpose:**

The purpose of this study was to compare the mechanical axis, accuracy of component positioning, and polyethylene liner thickness between robot-assisted total knee arthroplasty (TKA) and conventional TKA.

**Methods:**

From July 2020 to December 2020, 154 TKAs were performed in 110 patients with Kellgren-Lawrence grade IV varus knees using a robot-assisted system (MAKO group). Additionally, 110 propensity score-matched patients who had undergone primary conventional TKA were chosen in a one-to-one ratio for the conventional group. Post-operative radiographs were used to evaluate mechanical axis and component coronal and sagittal positioning. The polyethylene liner thickness was investigated. The respective mean error values and outliers were obtained for the two study groups and were compared to determine the mechanical axis and the accuracy of the postoperative component positioning.

**Results:**

Patients in the MAKO group achieved better accuracy than those in the conventional group in terms of postoperative mean mechanical axis (1.9˚ vs. 2.8˚, *p* < 0.05), femur coronal inclination (91.2˚ vs. 91.8˚, *p* < 0.05), tibia coronal inclination (90.8˚ vs. 91.1˚, *p* < 0.05), and tibia sagittal inclination (90.7˚ vs. 91.7˚, *p* < 0.05). However, there was no difference between the two groups in polyethylene liner thickness.

**Conclusions:**

Robot-assisted TKA showed improved mechanical axis and higher accuracy of component positioning compared to the conventional TKA technique, with no significant difference in polyethylene liner thickness between the two groups. Long-term follow-up studies are needed to compare the clinical outcomes of robot-assisted TKA.

**Level of evidence:**

IV.

## Introduction

Total knee arthroplasty (TKA) is an effective surgical intervention for the treatment of end-stage osteoarthritis of the knee as it can decrease pain and improve function. Appropriate component positioning and joint line restoration are important in TKA to ensure maximal implant longevity and increased patient function, as malalignment of components can result in greater risk of implant failure [8, 11]. Advances in technology and surgery for improved accuracy of component alignment led to development and utilization of a robot-assisted TKA system [5, 14, 16].

The MAKO robotic system (Stryker, Mahwah, NJ) is a semi-active robot that was first used for unicompartmental knee arthroplasty (UKA) in 2008. Adaptation for TKA has been available since 2016 [2]. In the MAKO robotic system, it is possible to determine size and location of the implant preoperatively. Dynamic reference guidance and bone mapping are used intraoperatively, allowing real-time tracking of the limb; this technology can lead to more accurate component positioning and limb alignment [2, 12, 13].

Currently, several studies have investigated the accuracy of component positioning in MAKO-assisted UKA. However, limited data exist on MAKO-assisted TKA [9, 10, 15]; in particular, few studies have addressed the comparison of polyethylene liner thickness between MAKO-assisted TKA and conventional TKA. The prediction of component positioning and joint line restoration after TKA might provide insight to better manage patient expectations and implant longevity [4].

Therefore, the aim of this retrospective case–control study was to compare the mechanical axis, accuracy of component positioning, and polyethylene liner thickness between MAKO-assisted TKA and conventional TKA. Our hypothesis was that MAKO-assisted TKA would result in better mechanical axis alignment and higher accuracy of component positioning and polyethylene liner thickness compared to conventional TKA.

## Materials and methods

The design and protocol of this retrospective study were approved by the institutional review board (IRB) of our hospital, and the requirement for informed consent was waived due to the retrospective nature of this study (IRB number: 116655–01-202,102–01).

The MAKO robot-assisted TKA system was introduced to our hospital in June 2020. Between July 2020 and December 2020, a consecutive series of 162 primary TKAs was performed for 116 patients at our hospital using the MAKO robotic system. Patients with preoperative Kellgren-Lawrence grade IV varus knee were included, and those with previous knee surgery history, body mass index (BMI) > 28, mechanical axis > varus 20°, valgus malalignment, or preoperative posterior tibia slope > 10° were excluded. The final cohort consisted of 90 females (127 knees) and 20 males (27 knees) (MAKO group). A total of 44 patients (40.0%) of the MAKO group underwent staged bilateral procedures every week. A group of 110 age-, sex-, body mass index-, and diagnosis-matched patients who underwent conventional primary TKA between January 2020 and December 2020 at our hospital were used as the control group and were propensity score-matched in a one-to-one ratio. In total, 154 TKAs were included in 110 patients (20 males and 90 females) (conventional group). Demographic data of age, sex, body mass index, and initial diagnosis were obtained by reviewing medical records (Table 1).

**Table 1:** Demographics of patients

	MAKO group	Conventional group	*P-*value
Cases (patients)	154 (110)	154 (110)	
Age (years)	70.8 ± 6.1	70.7 ± 6.3	n.s
Gender (Male: Female)	20: 90	20: 90	n.s
Body mass index (kg/m^2^)	24.8 ± 1.9	24.7 ± 2.1	n.s
Diagnosis, n (%)			n.s
Osteoarthritis	152 (98.7)	152 (98.7)	
Osteonecrosis	2 (1.3)	2 (1.3)	

*n.s.* not significant

All operations were performed at our hospital by a single experienced surgeon for the MAKO and conventional groups. All patients were treated using a posterior-stabilized Triathlon total knee prosthesis (Stryker Orthopedics, Mahwah, NJ, USA). Preoperative planning targets were neutral alignment and polyethylene thickness of 9 mm in both groups. All knees were exposed with a standard anterior midline incision via medial parapatellar approach, and the patella was everted laterally. The anterior cruciate ligament was resected off the femoral notch and tibia insertions, and the posterior cruciate ligament was removed from the notch. In the conventional TKA procedure, the intramedullary canal was accessed by drilling a hole located about 1 cm anterior to the center of the intercondylar notch. Femoral alignment guide was inserted into the intramedullary hole. After setting the instrument to the desired angle (valgus 6°) and placing it in the appropriate notch, distal femoral resection was conducted. The assembly flush on the resected distal femur was positioned, and proper femoral size and rotation (based on transepicondylar axis) were determined. The remaining four femoral bone resections and box cutting were performed. After placing the proximal rod of the tibial extramedullary resection guide at the center of the tibia and adjusting a 3° cutting block tilted by placing one finger at the tubercle of the proximal tibia and two fingers at the ankle, tibial cuts with a 0–1° posterior slope in the sagittal plane were made. Stability and alignment were assessed using the trial components. All implants were inserted with cement. In the MAKO-assisted TKA procedure, prior to surgery, a preoperative CT scan was performed and incorporated with the robotic software to identify optimal implant size and positioning. After the tracking arrays and check points were positioned on the femur and tibia, robot landmark calibration and bone registration and verification were performed by the probe to identify actual femoral and tibial bone position and limb alignment. The ‘Ligament Balancing’ workflow (Pre-Resection Balancing) applied proper tension to the knee joint in extension and flexion gaps. An appropriate implant position and orientation set per gap balancing was defined and saved in the robot system following the surgeon’s approval. The robotic arm saw performed distal femur, anterior cortex, posterior condyle, anterior chamfer, and posterior chamfer resection. Tibial resection was performed within virtual boundaries set by the robot to protect the soft tissues. After box cutting, femoral trial assessment and tibial trial assessment were performed. The femoral and tibial implants were implanted using bone cement, and polyethylene liner was deemed appropriate and impacted into place.

**Fig. 1:**
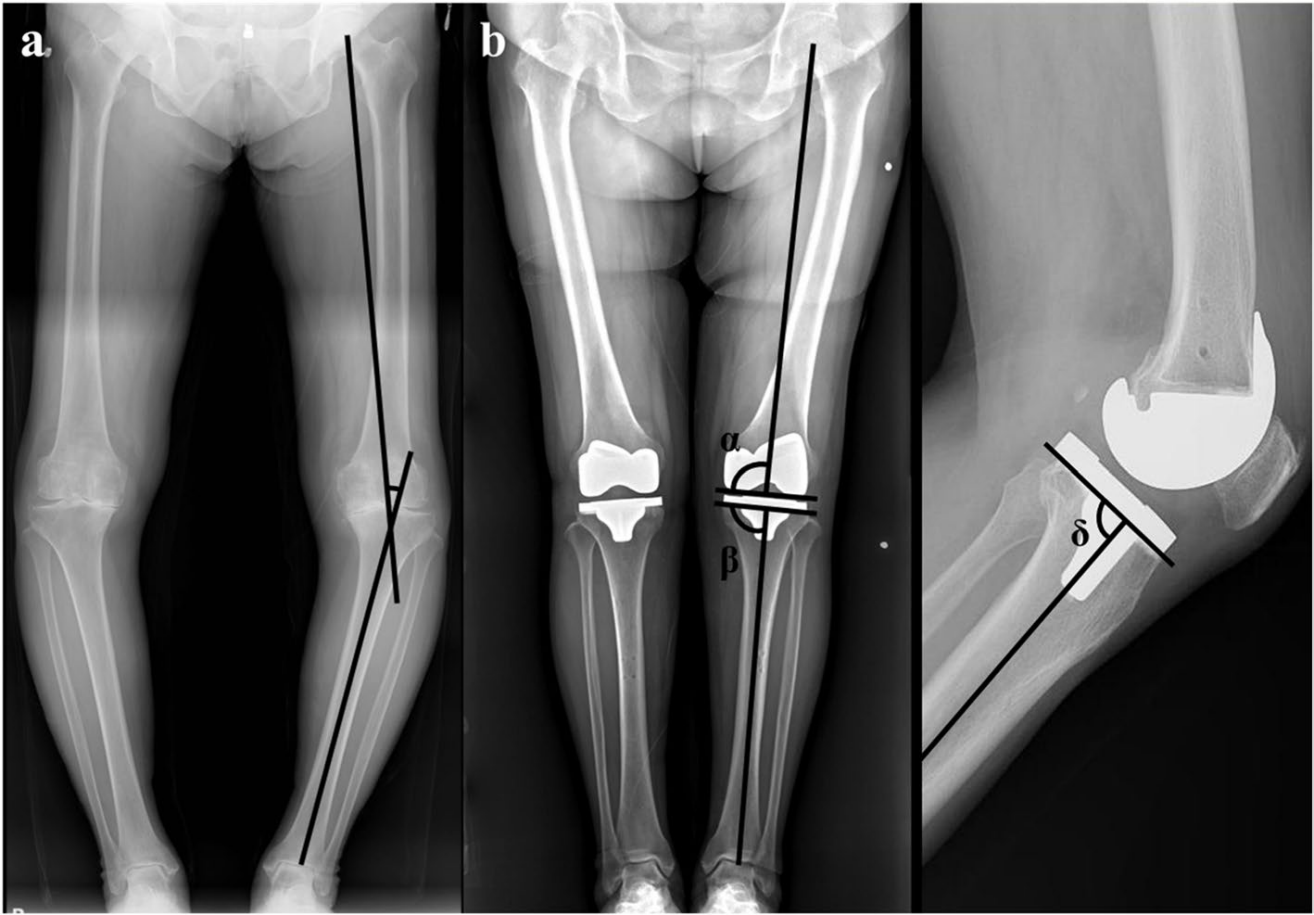
**a** The mechanical axis of the leg is the angle formed between the mechanical axis of the femur and tibia. **b** Radiologic measurement of femoral and tibial implants. α: coronal inclination of the femoral component with the mechanical axis of the femur, β: coronal inclination of the tibial component with the mechanical axis of the tibia, δ: sagittal inclination of the tibial component with the mechanical axis of the tibia

Patients underwent postoperative radiographic follow-up at two and six weeks; three, six, nine, and 12 months; and annually thereafter. We considered the 6-week radiographs the baseline for radiographic comparison. To assess the mechanical axis (hip-knee-ankle) and femur and tibial coronal positioning (varus/valgus, α, β), standing anteroposterior radiographs of the bilateral lower extremities were used, and to assess tibia sagittal positioning (posterior/anterior slope, δ), lateral radiographs were used; all radiographs were evaluated by two independent observers [6] (Fig. 1). Also, polyethylene liner thickness was investigated. Outliers were defined as a measured angle exceeding 3 degrees from neutral alignment on each radiograph and liner thickness more than 16 mm. The mean error value was obtained for each study group and the values were compared to determine the accuracy of the postoperative component positioning and the polyethylene liner thickness.

The propensity score matching method using age, sex, body mass index, and diagnosis was used retrospectively to obtain a comparable control group. Differences in variables between the two groups were evaluated using Mann–Whitney rank test. The data are shown as mean ± standard deviation. Interclass correlation coefficients were calculated using SPSS version 20 and were applied to determine the correlation of the measurement between the two independent observers. Significance was set at *p*-value < 0.05 for all analyses.

## Results

The postoperative mean mechanical axis was 1.9˚ in the Mako group and 2.8˚ in the conventional group (*p* < 0.05). Patients in the MAKO group achieved better accuracy than those in the conventional group in terms of postoperative femur coronal (α), tibial coronal (β), and tibial sagittal inclination (δ) (*p* < 0.05) (Table 2). The number of postoperative outliers in terms of mechanical axis (> 3˚) was 28 (18.2%) in the Mako group and 55 (35.7%) in the conventional group, respectively (*p* < 0.05). There was no significant difference in polyethylene liner thickness between the two groups (Table 2).

**Table 2:** Comparison of radiologic results and polyethylene liner thickness between the MAKO and conventional groups

	MAKO group (*n* = 154)	Conventional group (*n* = 154)	*P-*value
Preoperative (degree)			
HKA axis	8.9 ± 3.9	9.0 ± 2.8	n.s
Posterior slope	7.4 ± 1.9	7.5 ± 2.0	n.s
Postoperative (degree)			
HKA axis	1.9 ± 1.6	2.8 ± 1.5	< 0.05
α	91.2 ± 0.8	91.8 ± 1.2	< 0.05
β	90.8 ± 0.5	91.1 ± 0.8	< 0.05
δ	90.7 ± 0.6	91.7 ± 1.0	< 0.05
Postoperative outliers, n (%)			
HKA axis	28 (18.2)	55 (35.7)	< 0.05
α	5 (3.2)	22 (14.3)	< 0.05
β	0 (0)	2 (1.3)	n.s
δ	0 (0)	14 (9.1)	< 0.05
Polyethylene liner thickness, n (%)			n.s
9 mm	84 (54.5)	76 (49.3)	
11 mm	55 (35.7)	54 (35.1)	
13 mm	15 (9.8)	20 (13.0)	
16 mm	0 (0)	4 (2.6)	

*HKA* hip-knee-ankle, *α* coronal inclination of the femoral component, *β* coronal inclination of the tibial component, *δ* sagittal inclination of the tibial component, *n.s.* not significant

The interclass correlation coefficient (ICC) was between 0.75 and 0.90, which was within the 95% confidence interval, signifying good correlation between the measurements of the two observers (Table 3).

**Table 3:** Interclass correlation coefficient (ICC) between two observers at postoperative

	Intraclass correlation	
	Robotic TKA	Conventional TKA
HKA axis	0.841 (0.788 to 0.882)	0.839 (0.784 to 0.880)
α	0.751 (0.658 to 0.819)	0.853 (0.796 to 0.894)
β	0.880 (0.839 to 0.911)	0.875 (0.831 to 0.907)
δ	0.903 (0.869 to 0.929)	0.922 (0.894 to 0.943)

The ICC between two observers was within the 95% confidence interval. < 0.5: poor, 0.50–0.75: moderate, 0.75–0.90: good, > 0.90: excellent

*HKA* hip-knee-ankle, *α* coronal inclination of the femoral component, *β* coronal inclination of the tibial component, *δ* sagittal inclination of the tibial component

## Discussion

This case–control study aimed to compare the mechanical axis, accuracy of component positioning and polyethylene liner thickness between MAKO-assisted TKA and conventional TKA. The most important finding of this study was that MAKO-assisted TKA showed improved mechanical axis and higher accuracy of the component positioning compared to conventional TKA, with no significant difference in polyethylene liner thickness between the two groups.

Accurate implant positioning and mechanical alignment might provide improvement in the patients’ function and increased implant longevity. Three comparative studies on implant positioning after robot-assisted TKA with the MAKO system have been published to date [9, 10, 15]. Hampp et al. [9] reported that robot-assisted TKA showed significantly more accurate bone cuts and implant positioning compared to conventional TKA. Kayani et al. [10] demonstrated that robot-assisted TKA improved the accuracy of coronal and sagittal alignment in femoral and tibial components, joint line restoration, tibia slope, and limb alignment compared to conventional TKA. Sultan et al. [15] reported that mean postoperative posterior condylar offset ratio was higher in conventional TKA, and the number of patients who had postoperative Insall-Salvati Index outside of the normal range were higher in conventional TKA. These studies demonstrated that MAKO-assisted TKA was more accurate than conventional TKA in restoring appropriate mechanical axis and diminishing the number of outliers. In this study, MAKO-assisted TKA was associated with improved accuracy in limb alignment and component positioning compared to conventional TKA. However, the 35.7% (55/154) outlier rate for the mechanical axis in the conventional group seems quite high. It is thought to be due to variables such as femoral bowing rather than technical errors. Further studies are needed to determine whether such a high outlier rate of the mechanical axis will affect clinical prognosis.

Joint line restoration is vital in primary TKA, contributing to range of motion, mid-flexion stability, patellofemoral joint mechanics, and functional outcomes [1]. Figgie et al. [7] highlighted the importance of preserving the joint line within 8 mm to avoid complications of anterior knee pain, stiffness, and revision surgery. The use of thicker polyethylene liners is associated with elevation of the knee joint line. Berend et al. [3] performed a retrospective study using implant survivorship following 6070 primary TKAs and reported inferior implant survival rates in patients with thick liners. In this study, a polyethylene liner thickness of 9 mm was our target, but approximately 50% of both groups did not achieve this goal. This suggests that it is difficult to predict the ligament balance even in robot-assisted TKA. A significant difference was not observed between the two groups for polyethylene liner thickness (*p* = 0.321). However, our study noted 0% (0/154) liner outliers in MAKO-assisted TKA compared to 2.6% (4/154) outliers in conventional TKA. It is assumed that this can lead to a reduced incidence of knee complications and improved functional outcomes for patients undergoing MAKO-assisted TKA. This could be due to the relatively small sample size study and the high-volume knee surgeon.

MAKO-assisted TKA has several distinct disadvantages over conventional TKA. First, there is an additional radiation risk resulting from the preoperative CT scan that is not required in conventional surgery. Second, due to its relatively higher cost, the availability to patients of MAKO-assisted TKA is limited. Third, one of the possible adverse events during robot-assisted procedures is pin tract-induced periprosthetic fracture that does not occur in conventional procedures and that requires additional surgical management and hospitalization. Future research should consider whether this robot-assisted technology can justify the additional cost burden on the patient’s clinical outcomes and satisfaction.

This study has several limitations. First, the study was designed retrospectively and did not include clinical follow-up results. Second, because it was a single-center study involving patients treated by a single surgeon, accurate generalization of the outcomes might be limited. Third, we have not included assessment of femoral component placement as it is difficult to determine on lateral radiographs due to 7° anterior flange design of Triathlon. Because this design feature culminates in the potential to provide patients with a better fit, we do not measure the sagittal inclination of the femoral component. Lastly, our study used plain radiographs instead of postoperative CT scan to measure the accuracy of component positioning due to reduced patient economic burden.

In conclusion, MAKO-assisted TKA showed improved mechanical axis and higher accuracy of component positioning compared to the conventional TKA. In addition, a larger study and long term follow up would be needed to establish the advantages for patients’ clinical outcome and justification of cost.

## Data Availability

All data generated or analyzed during this study are included in this published article.
